# ß-Hydroxybutyrate Improves Mitochondrial Function After Transient Ischemia in the Mouse

**DOI:** 10.1007/s11064-022-03637-6

**Published:** 2022-06-08

**Authors:** Alina Lehto, Konrad Koch, Johanna Barnstorf-Brandes, Christian Viel, Marius Fuchs, Jochen Klein

**Affiliations:** 1 Department of Pharmacology and Clinical Pharmacy, College of Pharmacy, Goethe University of Frankfurt, Max-von-Laue-Str. 9, 60438 Frankfurt, Germany; 2 Institute of Legal Medicine, Kanton Basel, Basel, Switzerland

**Keywords:** Complex I, Complex II, Glucose, Microdialysis, Oxidative phosphorylation, Stroke

## Abstract

ß-Hydroxybutyrate (BHB) is a ketone body formed in high amounts during lipolysis and fasting. Ketone bodies and the ketogenic diet were suggested as neuroprotective agents in neurodegenerative disease. In the present work, we induced transient ischemia in mouse brain by unilaterally occluding the middle cerebral artery for 90 min. BHB (30 mg/kg), given immediately after reperfusion, significantly improved the neurological score determined after 24 h. In isolated mitochondria from mouse brain, oxygen consumption by the complexes I, II and IV was reduced immediately after ischemia but recovered slowly over 1 week. The single acute BHB administration after reperfusion improved complex I and II activity after 24 h while no significant effects were seen at later time points. After 24 h, plasma and brain BHB concentrations were strongly increased while mitochondrial intermediates (citrate, succinate) were unchanged in brain tissue. Our data suggest that a single administration of BHB may improve mitochondrial respiration for 1–2 days but not for later time points. Endogenous BHB formation seems to complement the effects of exogenous BHB administration.

**Supplementary Information:**

The online version contains supplementary material available at 10.1007/s11064-022-03637-6.

## Introduction

Cerebral ischemia has severe consequences including death and disability [1]. Drug treatment of cerebral ischemia, to this day, is unsatisfactory apart from the use of recombinant tissue plasminogen activator (rtPA). While the healthy brain almost exclusively uses glucose as energy substrate, ketone bodies such as ß-hydroxybutyrate (BHB) can substitute for glucose under certain conditions, e.g. in early life or during prolonged fasting [2]. When ketone bodies reach high (5–10) millimolar concentrations, up to half of the brain energy consumption can be supplied by ketone bodies [3, 4].

The ability of ketone bodies to energize the brain have led to a range of studies to elucidate if ketone bodies may have neuroprotective activity [5]. In brain ischemia, hyperglycemia is detrimental, whereas ketone bodies have significant benefits. Animal work has shown that fat-rich and ketogenic diets reduce the outomes of global ischemia and stroke [6]. In focal ischemia, diet-induced ketosis as well as the administration of BHB improved neurological function [7, 8]. Exogenous BHB also prevented neuronal death in models of Alzheimer’s and Parkinson’s disease [9, 10].

The mechanism of action of BHB in ischemia remains to be firmly established [11]. BHB has multiple activities in the brain, interacting with ion channels and inhibiting histone deacetylation [12]. BHB also has indirect antioxidative activity and inhibits neuroinflammation [13, 14]. Some evidence connects mitochondrial function to BHB´s actions. In the brain, BHB can be converted to acetoacetate in mitochondria, producing NADH and, by further cleavage, acetyl-CoA [12]. BHB administration can increase succinate concentrations, stabilize complex II activity and reduce reactive oxygen generation [8, 10]. 13C-labeled BHB, given to humans, was metabolized into glutamate and glutamine in the brain, a pathway mediated by the citric acid cycle [15]. In our hands, significant BHB formation was observed after stroke, an effect that was strongly stimulated in mice fed a fat-rich diet [16]. These finding led us to investigate mitochondrial function after transient ischemia and a single administration of BHB.

## Materials and Methods

### Chemicals

Chemicals were purchased from Sigma/Merck (Darmstadt, Germany) unless stated otherwise.

### Animals

Female CD-1 mice (29–32 g, Charles River) were used for the experiments. They were kept in standard cages, under 60% humidity, 22 °C temperature, and a 12 h-light/dark cycle. Food and water were available ad lib. The study was registered with the local animal committee (Regierungspräsidium Darmstadt). In accordance with GV-Solas guidelines, all procedures were designed to minimize the suffering of the experimental animals.

**Fig. 1:**
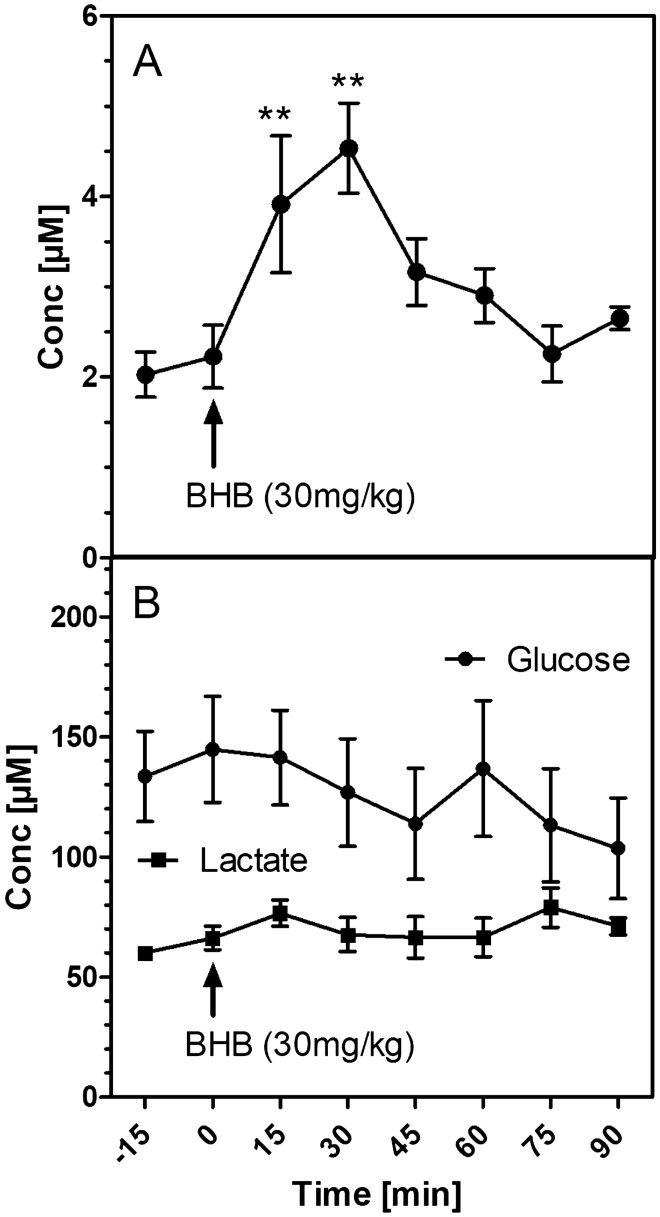
Changes of metabolite levels in hippocampal microdialysates after i.p. administration of 30 mg/kg sodium ß-hydroxybutyrate (BHB). **A** BHB; **B** glucose and lactate. Data are means ± SEM of six experiments. Statistics were calculated by repeated measures ANOVA and Dunnett’s multiple comparison test. **p < 0.01 vs. basal value at − 15 min

**Fig. 2:**
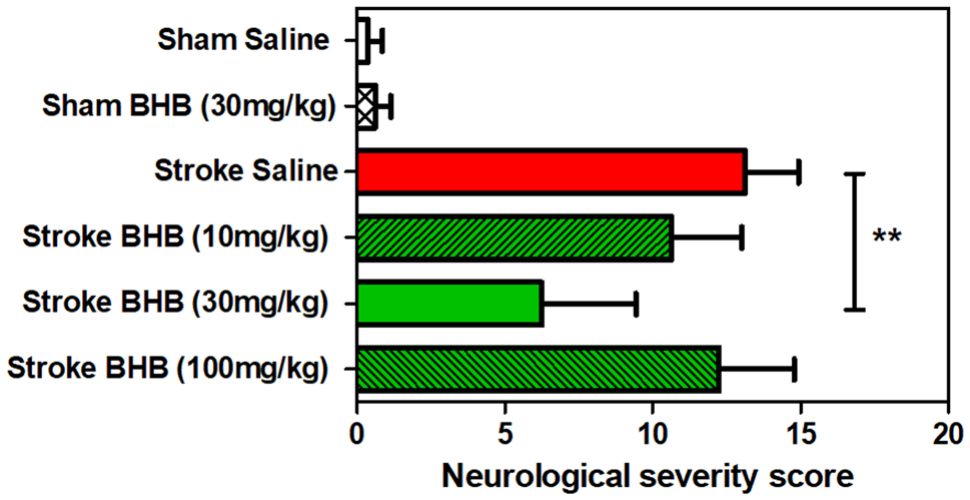
Effects of stroke and ß-hydroxybutyrate (BHB) on neurological outcome. Mice underwent transient cerebral ischemia for 90 min and were given saline or BHB (10–100 mg/kg) by i.p. injection immediately after reperfusion “Stroke Saline”; “Stroke BHB”). For sham-operated mice (“Sham Saline”; Sham BHB”), the carotid artery was prepared but not occluded. A battery of neurological tests (see text) were carried out one day later. Maximum score was 15. Data are expressed as means ± SEM of N = 8 experiments. Data were evaluated by one-way ANOVA followed by Tukey´s mulitpile comparison test. **p < 0.01

**Fig. 3:**
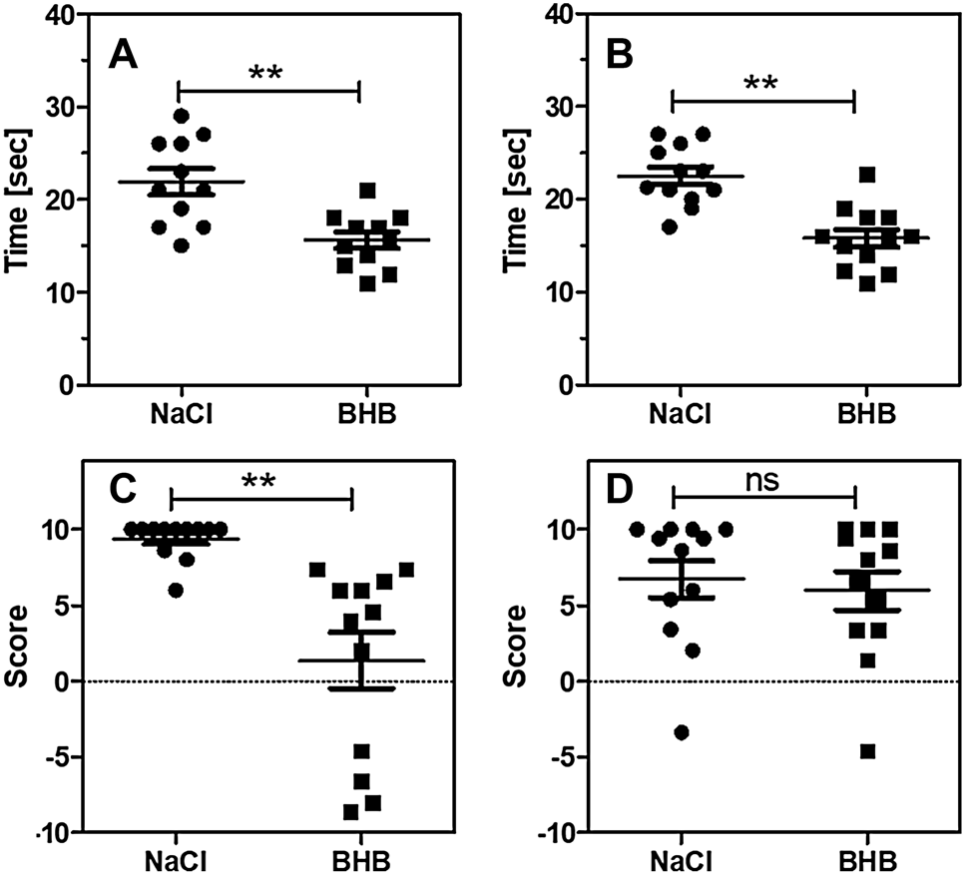
Effect of ß-hydroxybutyrate (BHB) on mouse motoric function. Mice underwent a transient cerebral ischemia for 90 min and were given saline or BHB (30 mg/kg) by i.p. injection immediately after reperfusion (“NaCl”; “BHB”). **A** Chimney Test after 24 h. This test measures the performance expressed in time (s) needed to exit a tube backwards (maximum value 120 s). **B** Chimney test after 72 h. **C** Corner Test after 24 h. This test determines the preferred side to leave a corner. A score of zero represents equal number of turns to both sides, a score of 10 indicates that the animal always turned contralaterally to the brain lesion. **D** Corner test after 72 h. Data are scatter box plots (means ± SEM are indicated) of 10–14 independent experiments. **p < 0.01 (t-test)

**Fig. 4:**
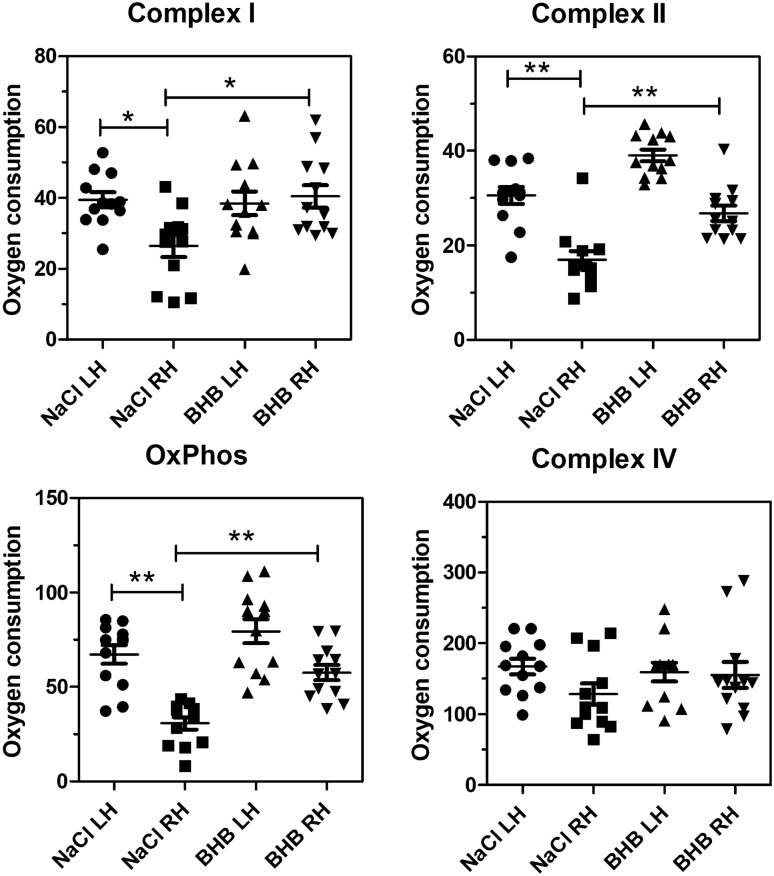
Oxygen consumption measured by respirometry 24 h after transient ischemia. **A** Complex I activity; **B** complex II activity; **C** oxidative phosphorylation (OxPhos); **D** Complex IV activity. Raw data were normalised to mitochondrial protein content. LH, left hemisphere; RH, right (ischemic) hemisphere. Statistical analysis: Data are scatter box plots (means ± SEM are indicated) of 12 independent experiments. Data were analysed by one-way ANOVA followed by Newman–Keuls post-test: **A** F_3,47_ = 4.86, p = 0.005; **B** F_3,47_ = 30.93, p < 0.001; **C** F_3,47_ = 18.77, p < 0.001; **D** F_3,47_ = 1.33, p = 0.28. *p < 0.05, **p < 0.01

**Fig. 5:**
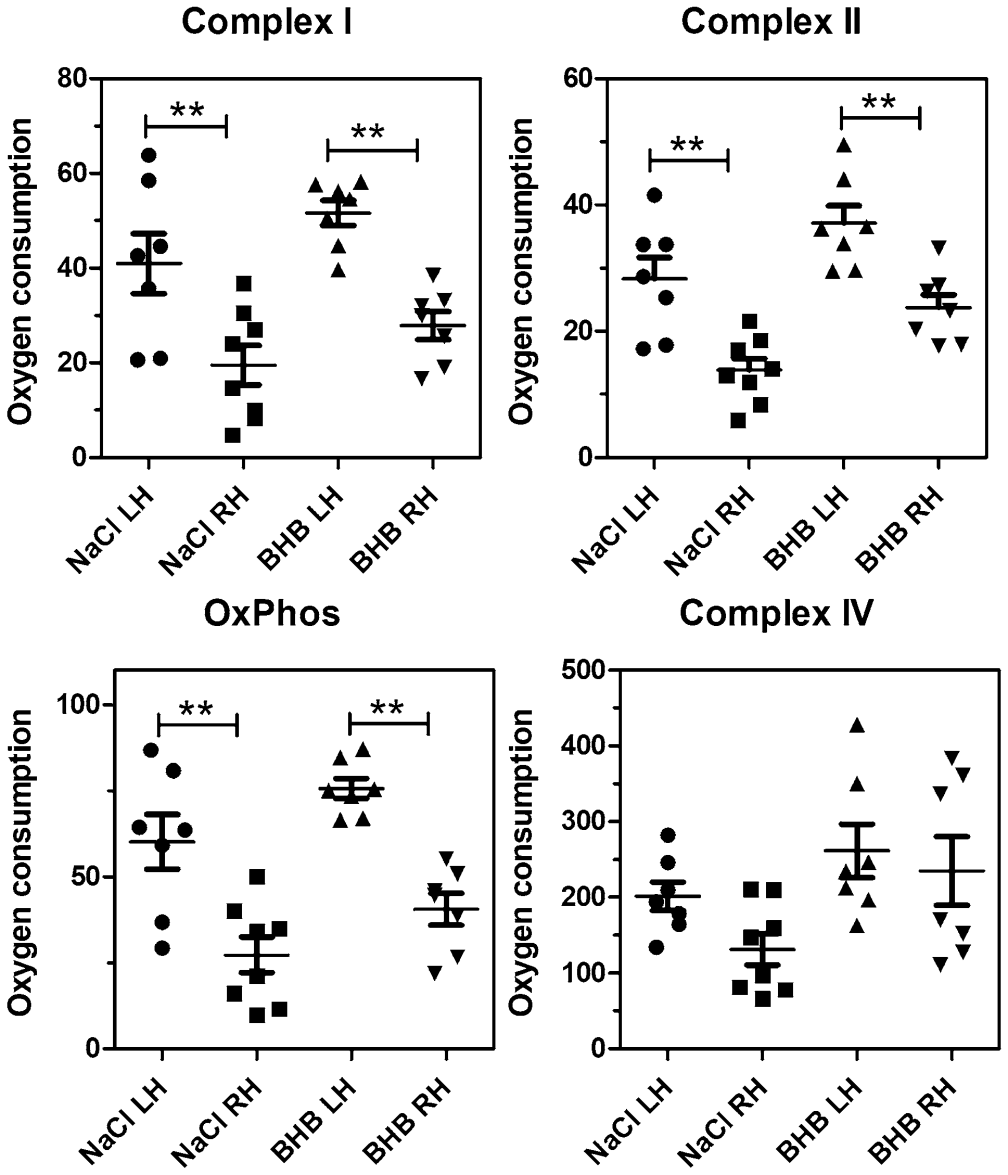
Oxygen consumption measured by respirometry 72 h after transient ischemia. **A** Complex I activity; **B** complex II activity; **C** oxidative phosphorylation (OxPhos); **D** Complex IV activity. LH, left hemisphere; RH, right (ischemic) hemisphere. Raw data were normalised to mitochondrial protein content. Statistical analysis: Data are scatter box plots (means ± SEM. are indicated) of 7–8 independent experiments. Data were analysed by one-way ANOVA followed by Newman–Keuls post-test: **A** F_3,28_ = 11.21, p > 0.001; **B** F_3,28_ = 14.79, p < 0.001; **C** F_3,28_ = 15.39, p < 0.001; **D** F_3,28_ = 3.37, p = 0.03. **p < 0.01

**Fig. 6:**
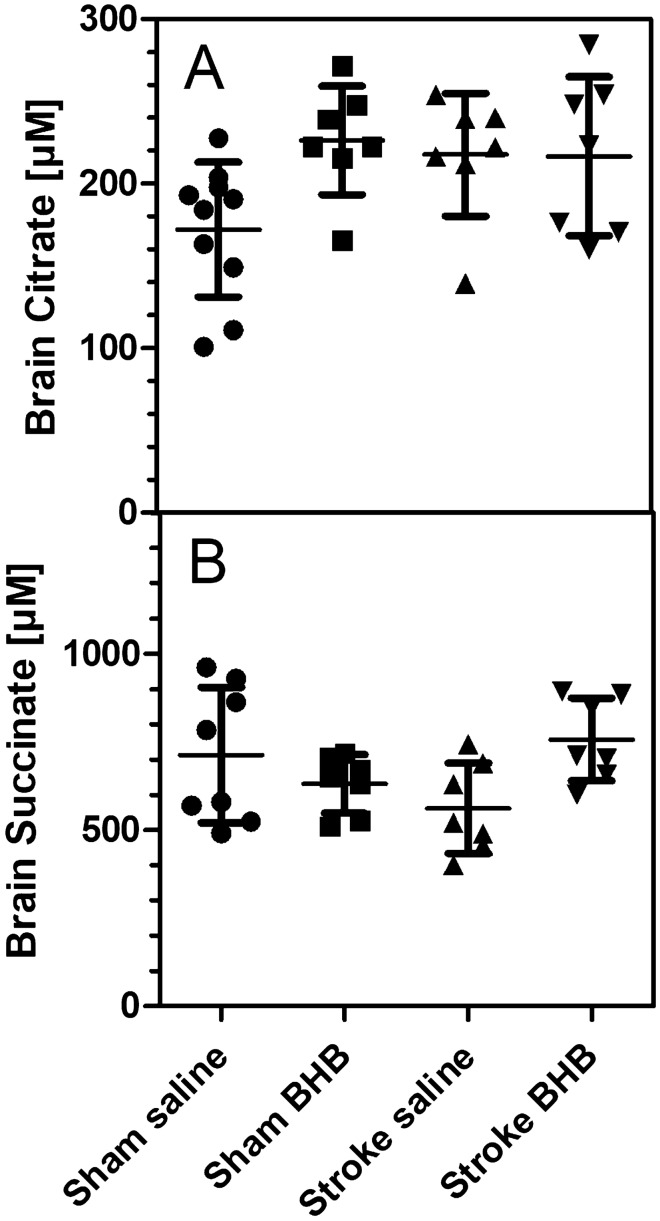
Concentrations of **A** citrate and **B** succinate in brain homogenate (ischemic hemisphere). Mice underwent transient cerebral ischemia for 90 min and were given saline or ß-hydroxybutyrate (BHB; 30 mg/kg) by i.p. injection immediately after reperfusion (“Stroke Saline”; “Stroke BHB”). For sham-operated mice (“Sham Saline”; Sham BHB”), the carotid artery was prepared but not occluded. Blood and brain samples were taken 24 h after BHB administration. Brain concentrations were calculated as µM assuming 80% brain water content. Data are given as means ± SD (N = 6–8). Statistics were calculated by One-way ANOVA and Tukey multiple comparison test: **A** F_3,27_ = 3.28; p = 0.04; **B** F_3,28_ = 2.78; p = 0.06

**Fig. 7:**
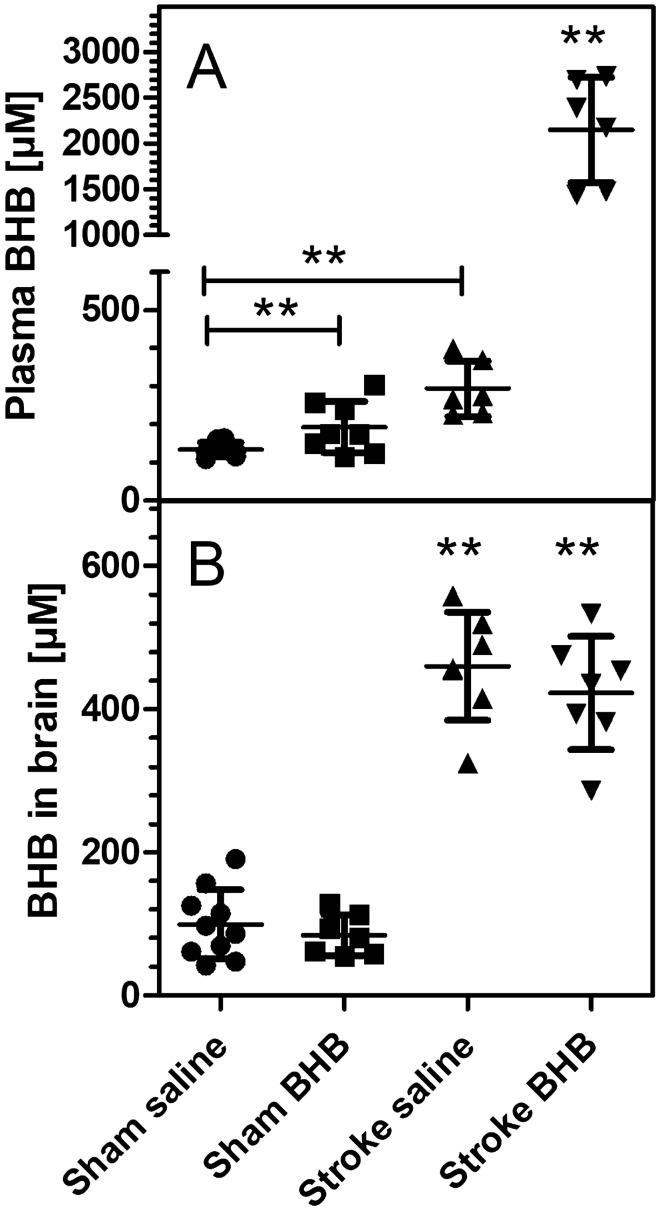
Concentrations of ß-hydroxybutyrate (BHB) in **A** blood plasma and **B** brain homogenate (ischemic hemisphere). Mice underwent transient cerebral ischemia for 90 min and were given saline or BHB (30 mg/kg) by i.p. injection immediately after reperfusion (“Stroke Saline”; “Stroke BHB”). For sham-operated mice (“Sham Saline”; Sham BHB”), the carotid artery was prepared but not occluded. Blood and brain samples were taken 24 h after BHB administration. Brain concentrations were calculated as µM assuming 80% brain water content. Data are given as means ± SD (N = 6–8). Statistics were calculated by One-way ANOVA and Tukey multiple comparison test: **A** F_3,27_ = 84.4; p < 0.001; **B** F_3,27_ = 86.3; p < 0.001. **p < 0.01 vs. Sham Saline

Mice were randomized to study groups using a computer program for random number generation. In total, 173 mice were used for this study. 24 experiments could not be followed through because of surgical problems (insufficient blockade of the MCAO, continuous bleeding during reperfusion), and the mice had to be sacrificed. In 17 experiments, analytical problems caused a failure to obtain data (lack of perfusion in the microdialysis probe, problems during sample work-up and GC–MS measurements). Thus, the results shown in Figs. 1, 2, 3, 4, 5 and 6 were obtained from 132 successful experiments (with an average of eight experiments per group). Separate experiments were performed for the generation of figures, except results in Figs. 6 and 7 that were from the same group of animals.

### Microdialysis Experiment

For surgery, animals were anesthetized with isoflurane (induction dose 5%, maintenance dose 2% v/v) in synthetic air (Air Liquide, Düsseldorf, Germany). Self-constructed, Y-shaped, concentric dialysis probes with a molecular weight cut-off of 10 kDa were stereotaxically implanted into the hypothalamus with the following coordinates (from bregma): AP − 1.5 mm, L + 0.5 mm, DV − 3.8 mm according to [17]. Glass ionomer eluting cement (PermaCem Smartmix Dual, Dental Milestone, Hamburg, Germany) was used to fix the probe on the skull (for further details, see [18]). Probes were implanted at least 18 h before each experiment to allow recovery to stabilize [19]. Microdialysis was performed on the next day with a perfusion fluid (aCSF) containing 147 mM NaCl, 4 mM KCl, 1.2 mM CaCl_2_ and 1.2 mM MgCl_2_. The perfusion rate of the microinjection pump was 2 µL/min. The collection intervals were 15 min. Data are given as absolute levels not adjusted for probe recovery.

Glucose and lactate concentrations in microdialysates were determined by a colorimetric method (530 nm) using an ISCUSflex Microdialysis Analyzer (M dialysis AB, Solna, Sweden).

### Transient Middle Cerebral Artery Occlusion (t-MCAO)

The procedure was performed as previously described [20, 21]. Briefly, mice were anesthetized using isoflurane (2% in synthetic air), their body temperature was kept constant using a thermostatic device, and buprenorphine (0.1 mg/kg i.p.) was injected 15 min before performing surgery (this injection was repeated 8 h later). After a paratracheal incision, a silicon suture (Doccol®, Redlands, California; size 6–0) was inserted into the A. carotis communis and advanced into the middle cerebral artery (MCA), where the silicon top of the filament blocked perfusion of the vessel. Cerebral blood flow was monitored with a laser-Doppler monitoring device (Moor Instruments, Devon, UK) to ascertain ischemia (< 15% of blood flow vs. basal). After 90 min, the suture was removed to allow reperfusion. Mice were sacrificed under isoflurane anesthesia either after 60 min or after 1, 3 or 7 days.

### Behavioral Experiments

Neurological deficits were determined by behavioral testing in the morning before surgery and 24 h after MCAO. The “Chimney test” (modified from [22]) was performed for each mouse three times before and after surgery. A mouse was placed head first at the entry of a tube (200 mm long and 40 mm diameter). When the mouse reached the bottom of the tube, the tube was raised to an angle of 45 degrees. All mice reacted by walking backwards. The time needed to climb out of the tube was measured for a maximum of 120 s. The “Corner test” was used as described [23]. Mice were placed in a corner (30° angle) and the chosen sides to leave the corner were counted. Each mouse was tested for one trial (maximum time 120 s) before and after surgery. The laterality index (LI) was calculated: (left turns–right turns)/total number of turns [24]. After 24 h, we also calculated a neurological score from animal behavior. Details of the scoring procedure are given in the Suppl. Table 1.

### High-Resolution Respirometry in Isolated Mitochondria

After decapitation, the brain was immediately dissected from the skull, the cerebellum was removed and the brain divided into hemispheres. From each hemisphere the frontal part of the brain (≈100 mg) was separated and homogenized in 2 mL MiR05. In addition, a protease inhibitor cocktail (PI) was added to the medium (cOmplete Tablets EASY pack, Roche, Mannheim, Germany). The homogenate was centrifuged twice to remove all cell debris (1400×*g*, 7 min, 4 °C). The purified supernatant was then centrifuged again (10,000×*g*, 5 min, 4 °C), the resulting pellet containing the mitochondria was resuspended in 1000 µL MiR05 + PI and centrifuged once again (1400× *g*, 3 min, 4 °C). Finally, the supernatant was centrifuged one more time (10,000×*g*, 5 min, 4 °C) and the pellet resuspended in 250 µL MiR05 + PI.

Mitochondria from ischemic and contralateral hemispheres were put into parallel chambers of the respirometer. Each chamber was filled with 2.4 mL MiR05 medium according to manufacturer’s instructions and kept at 37 °C with constant stirring (750 rpm). After 30 min equilibration and subsequent air calibration, 80 µL of the mitochondrial suspensions were injected into the closed chamber. The remaining mitochondria were frozen in liquid nitrogen for protein determination with the Bradford assay. After equilibration, a solution containing pyruvate (5 mM) and malate (1 mM), two substrates linked to complex I (CI), was injected into the chamber (LEAK-state, non-phosphorylating resting state). Then, ADP (2 mM) was added to stimulate oxidative phosphorylation (OXPHOS; ADP-stimulated and CI-linked respiration). To induce the full ADP-stimulated respiration, succinate (10 mM), a CII-linked substrate, was injected (OXPHOS capacity). To verify the integrity of the outer mitochondrial membrane, cytochrome c (10 µM) was added; mitochondria whose respiration increased by more than 15% upon cytochrome c addition were discarded. The maximum capacity of the electron transfer system (ETS) was determined by the stepwise titration of the uncoupler FCCP (state E). To see the isolated CII respiration, the complex I inhibitor rotenone (2.5 µM) was added (CII-linked substrate state, uncoupled). After inhibition of complex III by antimycin A (2.5 µM), the residual oxygen consumption (ROX) remains, which is used to correct the mitochondrial respiration states. Ascorbate (2 mM) and tetramethyl-phenylendiamine (TMPD, 0.5 mM) are artificial electron donors that induce maximum cytochrome c- oxidase (complex IV, CIV) respiration by reducing cytochrome c. Ascorbate regenerates TMPD and is injected first. At the end of the experimental run CIV is inhibited by a high concentration of sodium azide (120 mM). The chemical background as well as ROX remains. To obtain the CIV activity this value has to be subtracted from the total measured oxygen flux (for further details, see [25]).

### Analytical Measurements

Blood plasma and brain samples were harvested immediately after decapitation of mice, frozen in liquid nitrogen and stored at− 80 °C until metabolites were measured by GC–MS. Brain homogenates were extracted using Folch’s procedure, the aqueous supernatant was dried under a stream of nitrogen, and the dry residues were derivatized with N,O-bis(trimethylsilyl) trifluoroacetamide (BSTFA) and trimethylchlorosilane (TMCS) (99:1). In plasma samples, proteins were precipitated by addition of methanol/water (9:1), centrifuged, and the supernatants were treated as described above.

Samples were measured on an HP-6890 Series GC-System (Hewlett Packard®, Palo Alto, California) coupled to an Agilent Mass Selective Detector 5973 (Agilent®, Waldbrunn, Germany) and an Agilent® Autosampler 7683. We used a VF-5MS capillary column (30 m × 0.25 mm inner diameter) (Varian Technologies®, Palo Alto, CA) with a silylated precolumn (5 m). After the qualitative analysis of the metabolites (spectra adjusted to N.I.S.T. database), we established single ion monitoring (SIM) parameters and used them for quantification of glucose, BHB, citrate, succinate, fumarate and malate. The calculations were done with internal and external standard methods.

### Statistical Procedures

If not indicated otherwise, data are presented as means ± SEM of N (number of animals). All data were tested for normal distribution by the Kolmogorov–Smirnov test (GraphPad Prism 5.03). Potential outliers (> 2 SD) were identified by the Grubbs test (https://www.graphpad.com/quickcalcs/grubbs). Sample size was calculated by the formula N = 2 SD^2^ × power index/delta^2^. Based on many years of experience, an SD of 20% was expected for metabolite measurements and a treatment effect of 25% was defined as goal of the study. The value for the power index (α = 0.05, two-sided; ß = 0.2; 80%) was taken from the book „Intuitive Biostatistics “ by Harvey Motulsky (Oxford University Press, 1995). Treatment effects on activity changes of mitochondrial respiration (Figs. 4, 5, Suppl. Figures 1–3) were compared using one-way analysis of variance (ANOVA; Prism 5.03; GraphPad Software, La Jolla, CA, USA) with Newman–Keuls post-test for multiple pair-wise comparisons. To compare means between two groups we used unpaired Student’s t-test (Fig. 3). P-values < 0.05 were considered to be statistically significant. All data were normally distributed, and no outliers were detected.

This is an exploratory study using mitochondrial parameters and levels of energy metabolites as major outcome variables. The experimenter was blinded to the animal groups during the measurements of corner and chimney tests. Apart from that, no blinding was performed in this study.

## Results

### Microdialysis Study

We first investigated whether exogenously applied ß-hydroxybutyrate (BHB) reaches the brain. The extracellular concentration of BHB in the brain was in the low micromolar range (Fig. 1A). After injecting 30 mg/kg of BHB (a dose that gave optimal results in the behavioral study, see below) the BHB level in the brain approximately doubled within 15–30 min, then returned to baseline. The levels of glucose and lactate (Fig. 1B) were not affected by the BHB injection.

### Neurological Outcome After Stroke

In the following experiments, transient cerebral ischemia was induced by unilaterally blocking the carotid artery for 90 min. After reperfusion, BHB (30 mg/kg) was injected intraperitoneally, and behavioral outcomes were observed 24 h later when the mice had recovered from surgery, anesthesia and pain medication. As shown in Fig. 2, sham-operated mice had no difficulties fulfilling the tasks (see Methods) but stroke induced a massive worsening of the neurological score. Mice showed motoric impairments and partial paresis, they had difficulties balancing on a round stick or a narrow beam. Importantly, the moderate dose of 30 mg/kg improved the score significantly whereas a lower (10 mg/kg) and a higher dose (100 mg/kg) had no effects. Therefore, the following experiments used the dose of 30 mg/kg BHB exclusively.

Figure 3 contrasts the performance of the stroked mice after 24 and 72 h. In the chimney test which requires considerable muscle strength, mice did not improve over time (Fig. 3A, B); a limited number of mice also showed poor performance after 7 days; data not shown). In BHB-treated mice, the time to leave the tube was reduced by approx. 5 s, a statistically significant effect at all time points. In the corner test which requires less muscular strength, untreated mice performed poorly after 24 h (Fig. 3C) but improved significantly after 72 h (Fig. 3D). BHB-treated mice performed best after 24 h, this was the only time point when BHB treatment showed a significant beneficial effect.

### Mitochondrial Activities

Based on previous work which identified BHB as a contributor to energy metabolism in the brain, we hypothesized that BHB administration may affect mitochondrial respiration. We first tested if BHB was effective when added to isolated mitochondria. As shown in Suppl. Figure 1, BHB was double as effective as pyruvate alone (p < 0.05). BHB-induced respiration was further increased after addition of malate (+ 79%) but pyruvate plus malate gave the highest signal, more than three times higher than BHB plus malate. Succinate also stimulated respiration significantly (six times more than pyruvate alone), but addition of malate did not further increase respiration (data not shown).

The following results were obtained in isolated mitochondria after induction of stroke. When mitochondria were isolated from mouse hemispheres 60 min after reperfusion, oxygen consumption in mitochondria from the ischemic hemisphere was less than 50% of that measured in the contralateral hemisphere (Suppl. Figure 2). Complexes I, II and IV were affected, and BHB administration was ineffective at this time point (Suppl. Figure 2). Figure 4 summarizes data obtained after 24 h of reperfusion. Here, the activity of the complexes I and II (and, consequently, oxidative phosphorylation) was further reduced in untreated animals and remained low at 72 h of reperfusion (Fig. 5). Importantly, after 24 h of reperfusion, BHB administration normalized complex I activity and increased complex II activity (Fig. 4 A, B) whereas complex IV total activity was not affected. At 72 h past reperfusion, the single administration of BHB still had beneficial effects, but the differences between saline-treated and BHB-treated mitochondria were no longer significant (Fig. 5 A–C). After one week, complexes I and II seem to recover but no effect of BHB could be seen (Suppl. Figure 3). It seems, therefore, that the single administration of BHB had a beneficial but transient effect on mitochondrial energy metabolism.

### Metabolite Concentrations in Brain Tissue

Since BHB effects were strongest at 24 h past administration, we measured energy metabolites in mouse brains 24 h after reperfusion. Brain levels of citrate and succinate are shown in Fig. 6 and were not affected by either ischemia or BHB administration. The same was true for the levels of malate and fumarate (data not shown). Finally, we determined the plasma and brain tissue levels of BHB (Fig. 7). Plasma BHB concentrations were 134 ± 20 µM in saline-treated, sham-operated mice and were increased significantly after BHB administration (193 ± 67 µM; Fig. 7A). Brain levels of BHB were not affected by BHB administration (Fig. 7B). After cerebral ischemia, however, BHB levels were increased in plasma (to 294 ± 73 µM, + 119% vs. sham-operated animals) and in brain (to 460 ± 76 µM; + 364% vs. sham-operated animals). Administration of BHB 24 h earlier caused a remarkable increase of plasma BHB (to 2.15 mM) and of brain BHB (to 423 µM) as illustrated in Fig. 7A and B. While plasma BHB was much higher in stroked mice after BHB administration (Fig. 7A), the brain tissue contents of BHB were high in stroked mice, irrespective of prior BHB application (Fig. 7B).

## Discussion

The present study dealt with BHB, a ketone body with neuroprotective properties. We first showed by microdialysis that BHB administration causes an increase of the BHB concentration in the extracellular space of the hippocampus. It is noteworthy that this increase was limited, likely due to extensive uptake of BHB by other organs as described previously [15, 26]. We then tested BHB’s effects when given as a single acute dose after 90 min of transient ischemia. 24 h later, the neurological scores indicated that BHB administration at 30 mg/kg attenuated the consequences of brain ischemia. It should be noted that neither a low nor a very high dose of BHB was able to affect the neurological outcome so that dosage seems to be important for beneficial effects. The reason why the high dose did not work remains unknown, the toxicity of BHB is considered low. In the following experiments, BHB was dosed at 30 mg/kg; at this dose, its beneficial effects at 24 h past ischemia partially disappeared at 72 h indicating that a single dose of BHB may have beneficial but transient effects. Of note, mice remained handicapped after 3 days in the challenging chimney test that requires a higher muscular effort, so that the effect of BHB was still visible after 3 days. In contrast, BHB only worked in the corner test after 24 h. This test requires less muscle strength and mice improved considerably within 3 days so that BHB was no longer effective. Clearly, the effects of repeated administration of BHB after ischemia should be investigated in future studies.

The present work focused on mitochondrial effects of BHB application. We first confirmed that BHB can be used for mitochondrial respiration in insolated mitochondria. We then tested mitochondrial effects 60 min past BHB administration. At this early time point, all mitochondrial complexes showed reduced respiration, but BHB administration had no effect. At 24 h past cerebral ischemia, complex IV activity had recovered, but it must be pointed out that in our assay, complex IV activity was measured ex vivo under optimum conditions of substrate supply, so that its activity is very high and does not reflect activity in situ. Complex I and II activities, and their combined activity which is reflected by “oxidative phosphorylation”, were significantly reduced after 24 and 72 h. After 24 h, BHB administration significantly improved mitochondrial respiration in these complexes. After 72 h, a minor effect was still visible but did not reach significance any more. It follows that the effects of the single BHB administration were transient, similar to the results found in behavioral assays. In other words, a similar time course was observed for BHB’s actions on mitochondrial respiration and on functional outcomes.

In a final series of experiments, we measured BHB levels in plasma and brain, and mitochondrial metabolites in brain tissue. 24 h after administration, plasma BHB levels were higher than in controls (by 44%). Brain BHB concentrations were unchanged in non-ischemic mice 24 h after BHB administration. Cerebral ischemia, however, caused ketosis in mice: Plasma levels rose to 292 µM after stroke and, 24 h after BHB administration, to 2.15 mM. Brain tissue levels reached values of 400–500 µM BHB, five times higher than in non-stroked mice. This massive increase of ketone bodies was also observed in our previous study [16] in which ketosis after stroke was particularly strong in mice kept on a fat-rich diet. In this earlier study, ketosis was prevented by propranolol, a blocker of adrenergic ß-receptors, and we hypothesized that the well-known activation of the sympathetic nervous system post stroke induced ketone body formation in liver, mediated by adrenaline [27]. In our present study, mice had been kept on standard diet, but nevertheless ketosis was visible in blood and brain. While BHB may also accumulate in the brain due to lack of metabolism in hypoxic conditions, in our hands BHB brain levels were higher after stroke than in plasma, at least in the stroke-saline group, and this finding could be explained by BHB synthesis in the brain. While most ketone bodies are synthesized in the liver, astrocytes have also been shown to produce ketone bodies [28, 29], and this pathway may be neuroprotective under conditions of hypoglycemia [30, 31]. We speculate that BHB accumulates in the brain due to slow further metabolism, possibly because of lack of oxygen and NAD^+^ in hypoxic conditions. Still, the functional tests show that some BHB is evidently metabolized and attenuates neuronal dysfunction, improving motoric function.

In summary, we confirm previous reports [7, 32] that ß-hydroxbutyrate has neuroprotective properties in cerebral ischemia. Several mechanisms of action have been proposed for BHB actions: Some work suggested an inhibition of neuroinflammation through HCA2 receptors [33, 34], other studies favored epigenetic mechanisms through histone deacetylase inhibition and reduction of reactive oxygen species (antioxidative mechanism) [35, 36]. While our study does not exclude these mechamisms, we suggest that BHB’s beneficial action is associated with the improvement of mitochondrial function. Earlier studies have also reported mitochondrial effects in animal models [36]. In one study, an increase of succinate was suggested to mediate BHB´s actions [10]. In our hands, total brain succinate levels were stable after BHB, but we cannot exclude dynamic local changes of citric acid cycle metabolites. Nevertheless, we suggest that in our model, BHB likely acted by improving complex I and II activities and therefore, mitochondrial function.

## Conclusion

ß-Hydroxbutyrate, a ketone body, can improve mitochondrial function and behavioral outcomes at 24 h when given immediately after transient cerebral ischemia in mice. The effect is dose-dependent and transient as improvements already disappear after 72 h. The potentially beneficial effects of a prolonged administration of BHB after cerebral ischemia should be investigated.

## Supplementary Information

Below is the link to the electronic supplementary material.

Supplementary file1 (TIF 3374 kb)

Supplementary file2 (TIF 13595 kb)

Supplementary file3 (TIF 13493 kb)

Supplementary file4 (PDF 405 kb)

## Data Availability

The data that support the findings of this study are available from the corresponding author upon reasonable request.
