# Supplementary figures and images for: ß-Hydroxybutyrate Improves Mitochondrial Function After Transient Ischemia in the Mouse
Source: Neurochem Res. 2022 Jun 8;47(11):3241–9. doi: 10.1007/s11064-022-03637-6 (PMC9546981; doi:10.1007/s11064-022-03637-6)

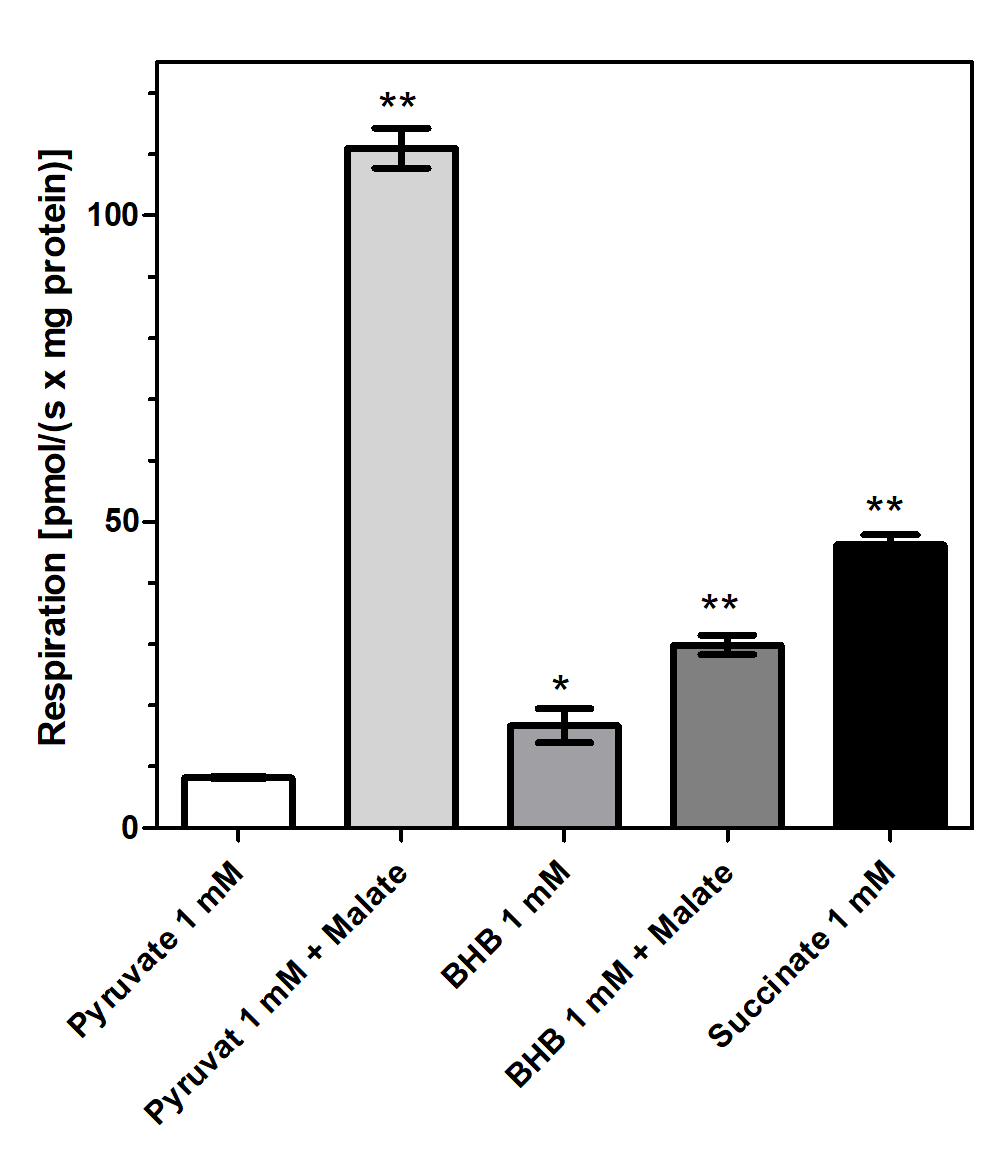

Supplement: Supplementary file 1 — Supplementary file1 (TIF 3374 kb) [file 11064_2022_3637_MOESM1_ESM.tif]

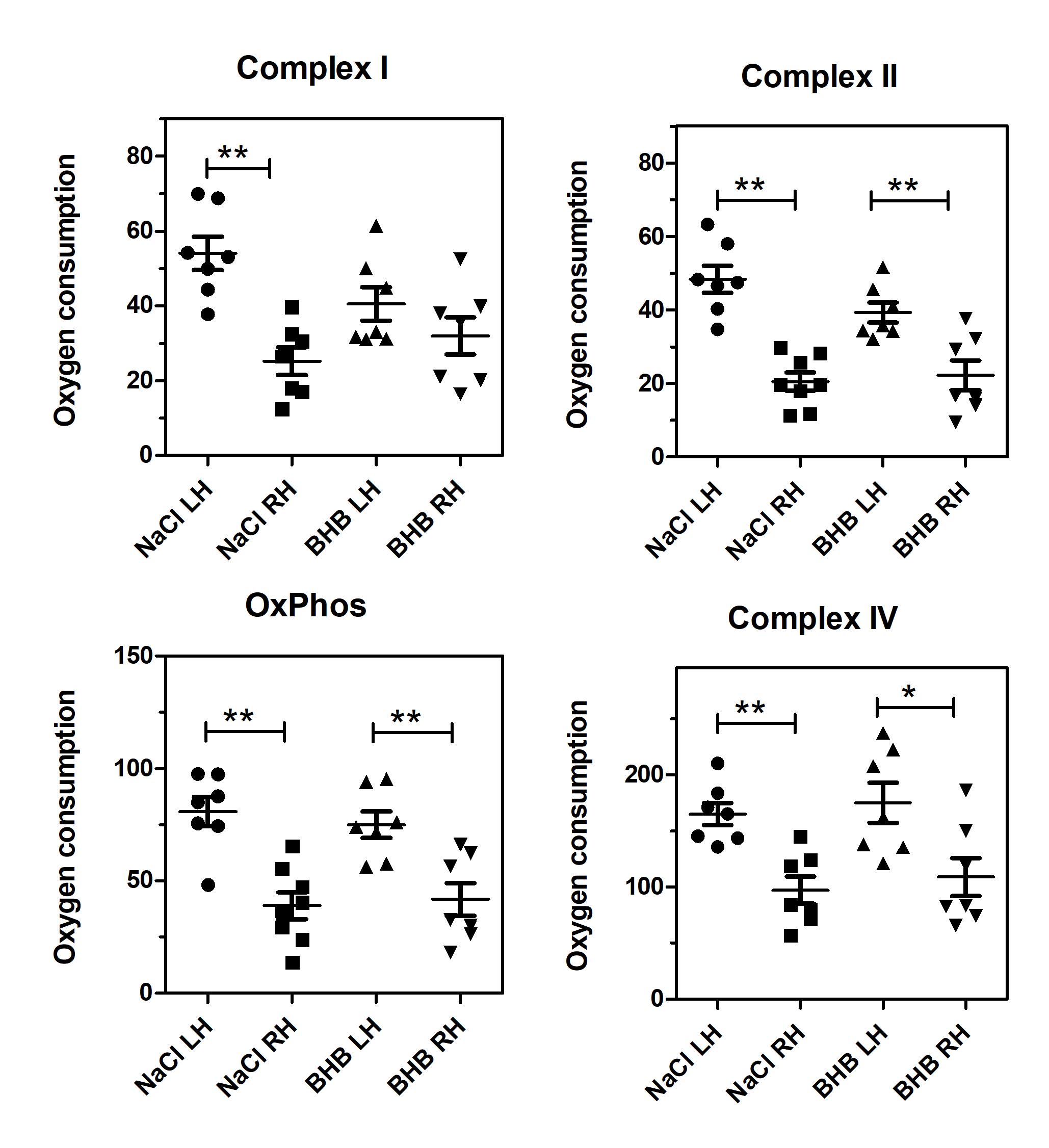

Supplement: Supplementary file 2 — Supplementary file2 (TIF 13595 kb) [file 11064_2022_3637_MOESM2_ESM.tif]

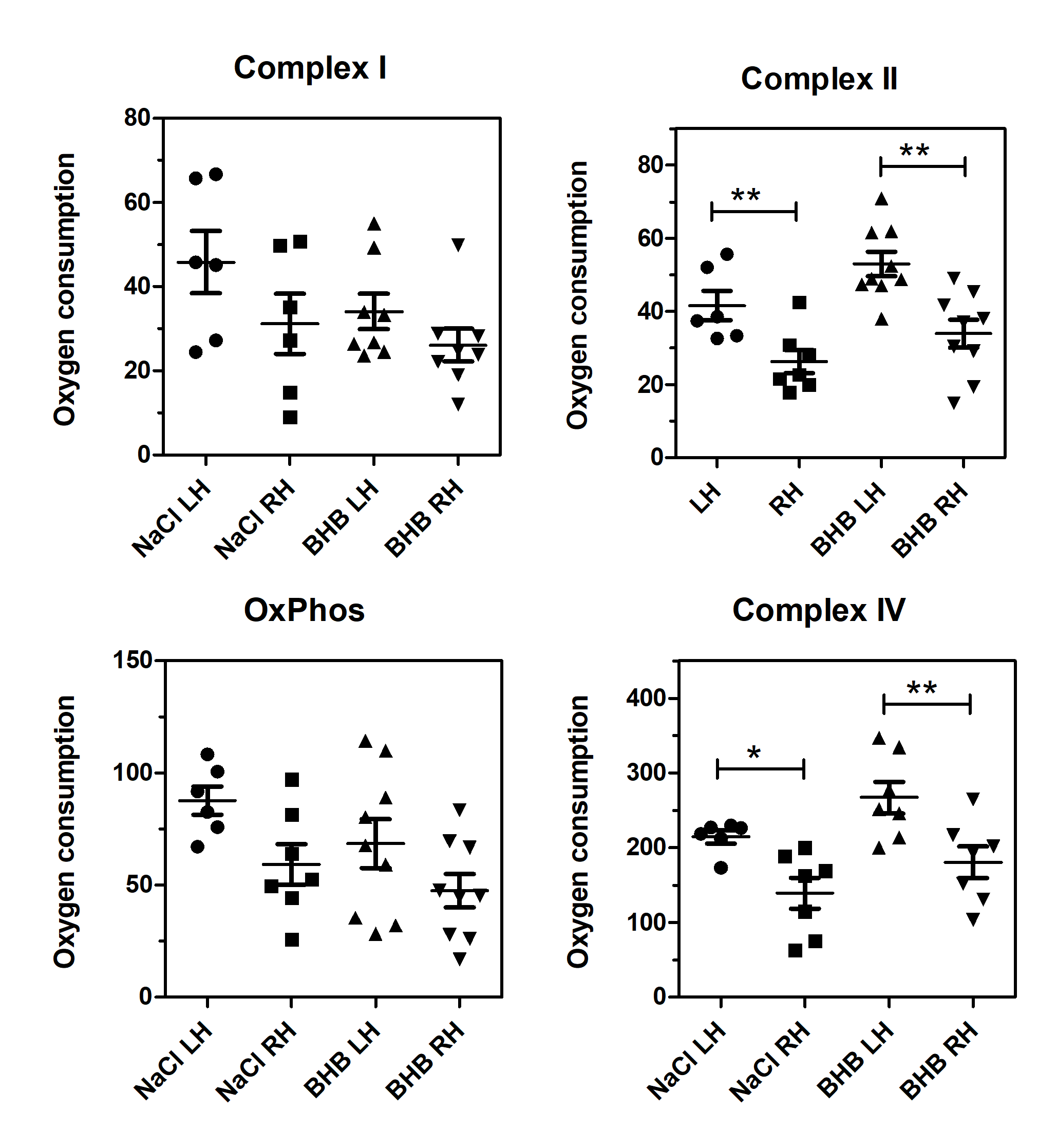

Supplement: Supplementary file 3 — Supplementary file3 (TIF 13493 kb) [file 11064_2022_3637_MOESM3_ESM.tif]
