# Supplementary material for: ß-Hydroxybutyrate Improves Mitochondrial Function After Transient Ischemia in the Mouse
Source: Neurochem Res. 2022 Jun 8;47(11):3241–9. doi: 10.1007/s11064-022-03637-6 (PMC9546981; doi:10.1007/s11064-022-03637-6)
Supplement: Supplementary file 4 — Supplementary file4 (PDF 405 kb) [file 11064_2022_3637_MOESM4_ESM.pdf]

Suppl. Table 1: Neurological Severity Score

Task	Description	Points	
		Success	Failure
Exit Circle	Ability and initiative to exit a circle of 30 cm diameter through a small hole in the wall (time limit 3 minutes).	0	1
Mono-/Hemiparesis	Paresis of upper and/or lower limb of the contralateral side to the intervention.	0	1
Straight Walk	Initiative and motor ability to walk straight once the animal is put on the floor.	0	1
Seeking Behavior	Alertness, curiosity and interest in the environment.	0	1
Beam Balancing	Ability to balance on a beam of 7 mm width for at least 10 seconds.	0	1
Round Stick Balancing	Ability to balance on a round stick of 5 mm diameter for at least 10 seconds.	0	1
Beam Walk: 3 cm	Ability to cross a 30 cm long beam of 3 cm width.	0	1
Beam Walk: 2 cm	Ability to cross a 30 cm long beam of 2 cm width.	0	1
Beam Walk: 1 cm	Ability to cross a 30 cm long beam of 1 cm width.	0	1
Motor Test	Raising the mouse by tail results in flexion of fore and hind paws with a head movement of < 10° to the vertical axis within 30 seconds.	0	1
Walking Test	Normal walk<br>Inability to walk straight<br>Circling towards the paretic side<br>Fall down to the paretic side	0	 1<br>2<br><br>3
Ear Test	Head shaking when inner ear lobe is touched	0	1
Chimney test	Ability to exit a chimney of 20 cm length backwards at 90° angle within 30 sec	0	1
